# Supplementary material for: Hiltonol Cocktail Kills Lung Cancer Cells by Activating Cancer-Suppressors, PKR/OAS, and Restraining the Tumor Microenvironment
Source: Int J Mol Sci. 2021 Feb 5;22(4):1626. doi: 10.3390/ijms22041626 (PMC7915988; doi:10.3390/ijms22041626)
Supplement: Supplementary file 1 [file ijms-22-01626-s001.pdf]

Supplementary Figures

Figure S1. FACS analysis of apoptosis after combinatorial treatments of lung cancer cells.

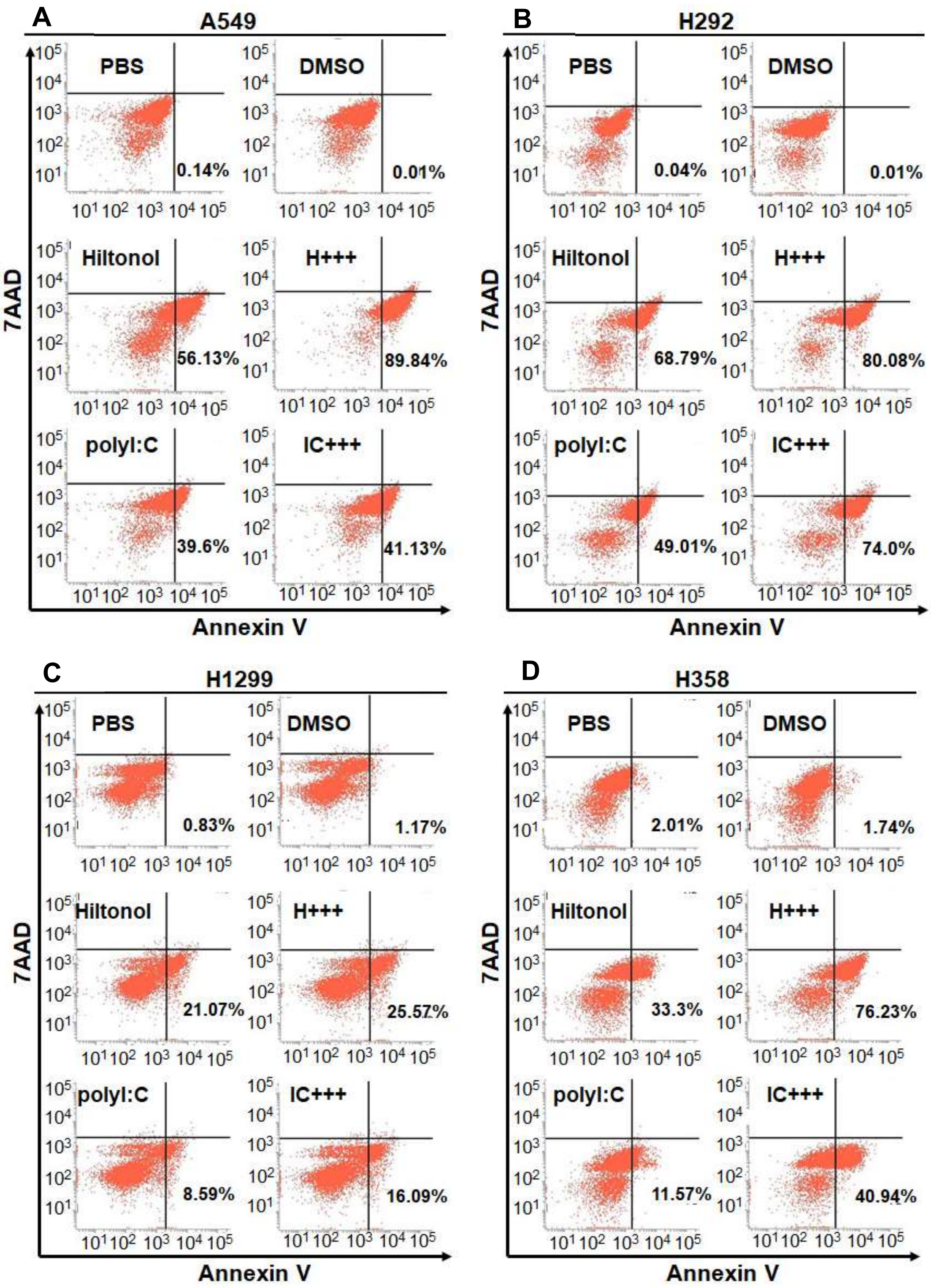

Figure S2. Representative microscopy images of lung cancer cell invasion.

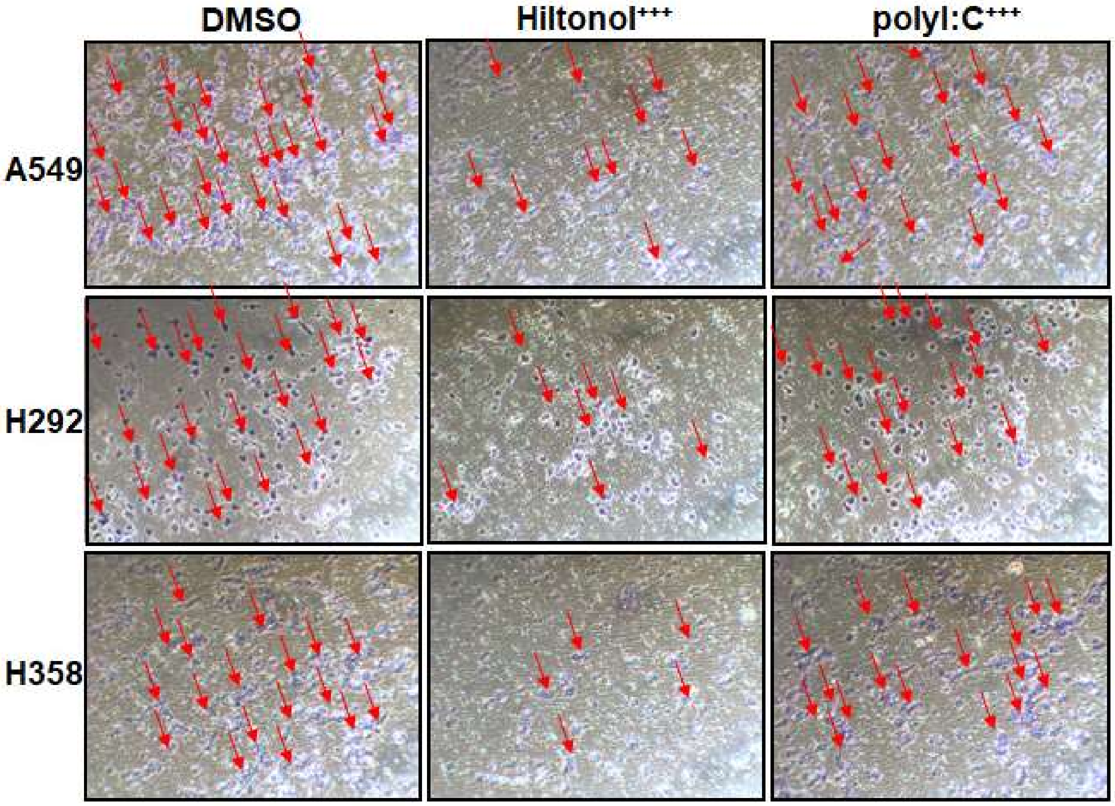

Figure S3. The efficacy of siRNA knockdown of PKR (and/or OAS) in H292 and H358 cells.

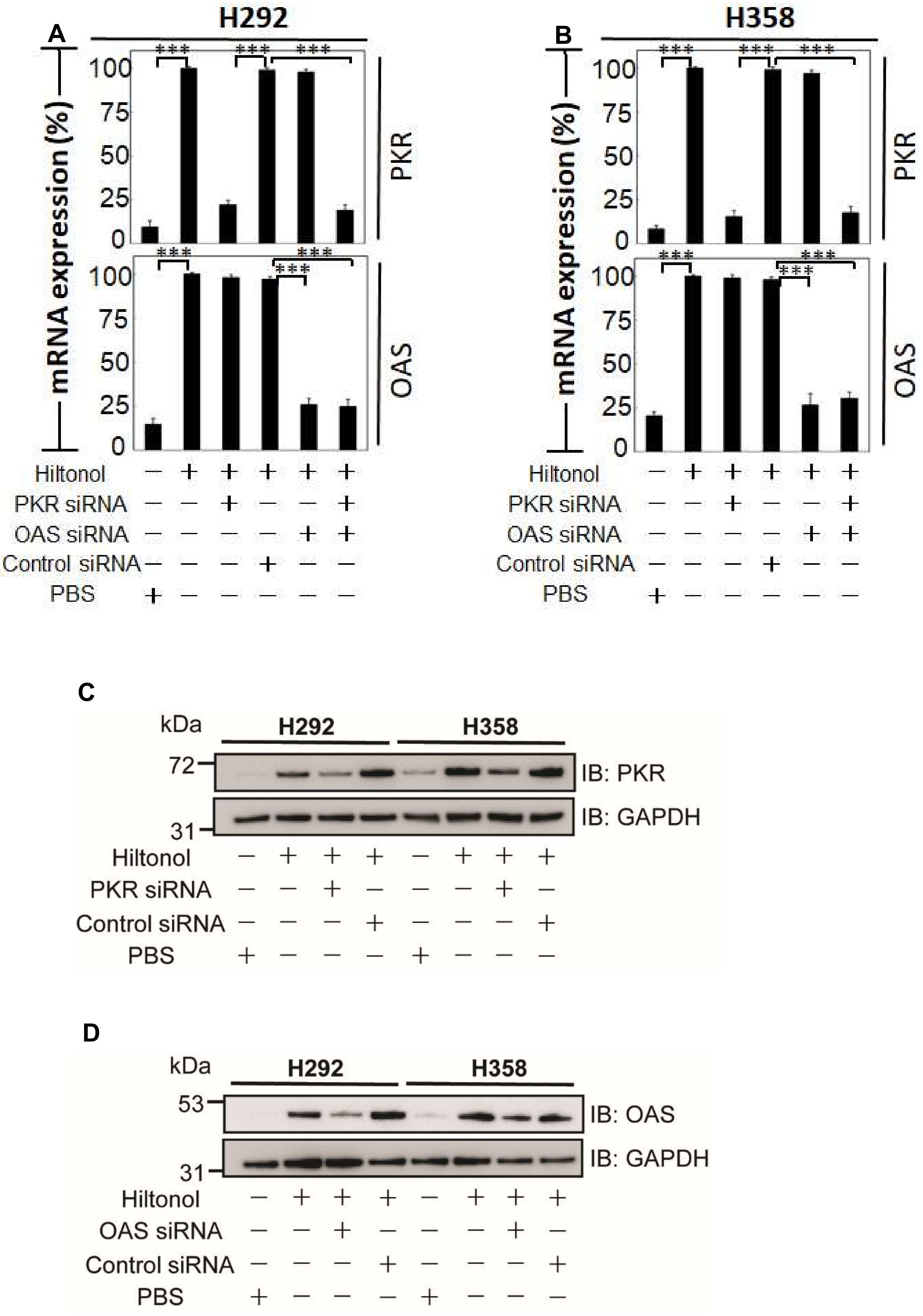

Figure S4. Controls of RNAi of PKR (and/or OAS) on cell viability and proliferation of H292 and H358 cells without Hiltonol treatment.

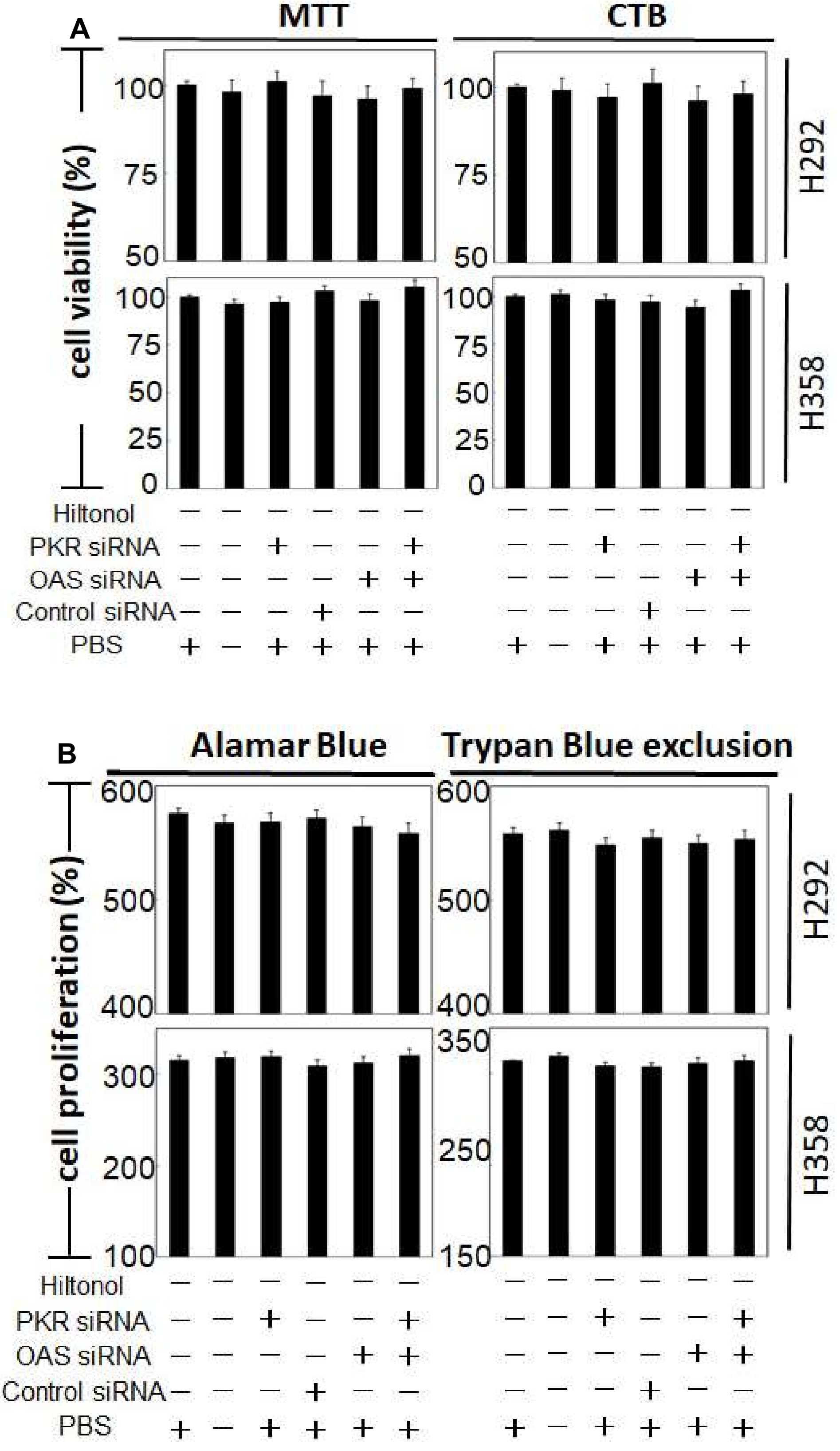

Figure S5. Clinicopathological parameters in paired primary human lung carcinoma tissues used for immunohistochemistry (IHC) analysis.

Patient	1	2	3	4	5	6	7	8	9	10	11	12	13	14
Diagnosis	AD	Acinar AD	AD	AD	AD	SCC	SCC	SCC	AD	AD	SCLC & AD	AD	AD	AD
Histologic type	Acinar AD	Acinar AD	AD	AD	AD	SCC	SCC	SCC	micropapillary AD	Acinar AD	AD	AD	Acinar AD	AD
Histologic grade	G1	G1	G2	G3	G2	G2	G2	G2	G3	G2	G3	G3	G1	G2
pT	T1b	T2a	T2a	T1a	T2a	T3	T3	T1a	T1b	T2a	T2a	T3	T2a	T3
pN	N0	N0	N0	N1	N1	N0	N0	N2	N2	N2	N2	N0	N2	N0
pM	MX	MX	MX	MX	MX	MX	MX	MX	MX	MX	MX	M1a	M1a	M1a
Stage	IA	IB	IB	IIA	IIA	IIB	IIB	IIIA	IIIA	IIIA	IIIA	IV	IV	IV
Age (year)	59	72	55	51	63	66	86	81	65	47	49	55	63	79
Gender (F/M)	F	F	M	M	M	M	M	M	F	M	F	M	F	F

Figure S6. Computational analysis reveals post-translational modification sites of PKR, OAS and IL-24.

A. PKR

```
1   MAGDLSAGFFMEELNTYRQKQGVVLKYQELPNSGPPHDRRFTFQVIIDGR 50
51  EFPEGEGRSKKEAKNAAAKLAVEILNKEKKAVSPLLLTTTNSSEGLSMGN 100
101 YIGLINRIAQKKRLTVNYEQCASGVHGPEGFHYKCKMGQKEYSIGTGSTK 150
151 QEAKQLAAKLAYLQILSEETSVKSDYLSSGSFATTCESQSNSLVTSTLAS 200
201 ESSSEGDFSADTSEINSNSDSLNSSSLLMNGLRNNQRKAKRSLAPRFDLP 250
251 DMKETKYTVDKRFGMDFKEIELIGSGGFGQVFKAKHRIDGKTYVIKRVKY 300
301 NNEKAEREVKALAKLDHVNIVHYNGCWDGFDYDPETSDDSLESSDYDPEN 350
351 SKNSSRSKTKCLFIQMEFCDKGTLEQWIEKRRGEKLDKVLALELFEQITK 400
401 GVDYIHSKKLIHRDLKPSNIFLVDTKQVKIGDFGLVTSLKNDGKRTRSKG 450
451 TLRYMSPEQISSQDYGKEVDLYALGLILAELLHVCDTAFETSKFFTDLRD 500
501 GIISDIFDKKEKTLLQKLLSKKPEDRPNTSEILRTLTVWKKSPEKNERHT 550
551 C
```

B. OAS

```
1   MMDLRNTPAKSLDKFIEDYLLPDTCFRMQINHAIDIICGFLKERCFRGSS 50
51  YPVCVSKVVKGGSSGKGTTLRGRSDADLVVFLSPLTTFQDQLNRRGEFIQ 100
101 EIRRQLEACQRERAFSVKFEVQAPRWGNPRALSFVLSSLQLGEGVEFDVL 150
151 PAFDALGQLTGGYKPNPQIYVKLIEECTDLQKEGEFSTCFTELQRDFLKQ 200
201 RPTKLKSLIRLVKHWYQNCKKKLGKLPPQYALELLTVYAWERGSMKTHFN 250
251 TAQGFRTVLELVINYQQLCIYWTKYYDFKNPIIEKYLRRQLTKPRPVILD 300
301 PADPTGNLGGGDPKGWRQLAQEAEAWLNYPCFKNWDGSPVSSWILLAGP 349
```

C. IL-24

```
1   MNFQQRLQSLWTLARPFCPPLLATASQMQMVVLPCLGFTLLLWSQVSGAQ 50
51  GQEFHFGPCQVKGVVPQKLWEAFWAVKDTMQAQDNITSARLLQQEVLQNV 100
101 SDAESCYLVHTLLEFYLKTVFKNYHNRTVEVRTLKSFSTLANNFVLIVSQ 150
151 LQPSQENEMFSIRDSAHRRFLLFRRAFKQLDVEAALTKALGEVDILLTWM 200
201 QKFYKL
```

Supplementary figure legends

Figure S1. FACS analysis of apoptosis after combinatorial treatments of lung cancer cells. Representative histograms of cell apoptosis assay (Annexin V and 7AAD double staining), including controls (PBS and DMSO). Four lung cancer cell lines are tested: **(A)** A549, **(B)** H292, **(C)** H1299 and **(D)** H358. Early apoptotic cells are indicated by Annexin V^{+}/$7AAD^{-}$ (shown as %). H^{+++}: Hiltonol+anti-IL6+stattic+AG490; IC^{+++}: polyI:C+anti-IL6+stattic+AG490.

Figure S2. Representative microscopy images of lung cancer cell invasion. 24 h after treatment of lung cancer cells with $Hiltonol^{+++}$, $polyI{:}C^{+++}$ or DMSO control, matrigel invasion assay was performed. $Hiltonol^{+++}$ efficiently suppressed lung cancer invasion. Three lung cancer cell lines are tested, including A549, H292, and H358. After another 24 h incubation, cells that had migrated from the upper to the lower side of the filter were imaged and counted with a light microscope (5 fields/filter). H^{+++}: Hiltonol+anti-IL6+stattic+AG490; IC^{+++}: polyI:C+anti-IL6+stattic+AG490.

Figure S3. The efficacy of siRNA knockdown of PKR (and/or OAS) in H292 and H358 cells. To examine PKR (or OAS) RNAi efficacy in H292 and H358 cells, PKR (or OAS)- sequence-specific siRNAs were transfected into the cells for 16 h, followed by Hiltonol (or PBS) treatment for 24 h. Then a quantitative real-time PCR for PKR and OAS was processed in: **(A)** H292 and **(B)** H358 cells. The mRNA expression levels from different treatments were compared with Hiltonol alone-treatment condition. All expression values were normalized against GAPDH as an endogenous control. Quantitative results are shown as mean ±SD (n=3). ***, $p<0.001$. Western blot immunodetections of: **(C)** PKR and **(D)** OAS were performed with H292 and H358 cells. GAPDH was used as a loading control.

Figure S4. Controls of RNAi of PKR (and/or OAS) on cell viability and proliferation of H292 and H358 cells without Hiltonol treatment. To measure the effects of PKR and/or OAS in lung cancer without Hiltonol treatment, at 24 h after transfection of PKR siRNA (or OAS siRNA or PKR+OAS double knockdown), NSCLC cells were treated with PBS for 48 h, followed by viability and proliferation studies. (**A**) Cell survival was assayed using MTT and CTB. For each treatment, cell counts were normalized with PBS-treated controls. (**B**) Cell proliferation was measured using Alamar Blue or Trypan Blue dye exclusion tests. For each condition, 48 h after treatment of PBS, cell counts were normalized with the corresponding 0 h treatment controls. Knockdown of either PKR or OAS, or PKR+OAS showed no significant

differences in cell viability or proliferation when the cells were untreated with Hiltonol.

Figure S5. Clinicopathological parameters in paired primary human lung carcinoma tissues used for immunohistochemistry (IHC) analysis. IHC clarified the physiological relevance of Hiltonol-mediated signaling in lung cancer in a total of 14-paired primary tissues (n=14 carcinoma; n=14 corresponding normal lung tissues). Samples are categorized into four groups based on staging, including: (1) IA/IB as initial stage (n=3 each of carcinoma and normal lung tissues); (2): IIA/IIB as early-to-middle stage (n=4 each of carcinoma tissues and normal lung tissues), (3) IIIA as middle-to-late stage (n=4 each of carcinoma and normal lung tissues) and (4): IV as late stage (n=3 each of carcinoma and corresponding normal lung tissues). Clinicopatholoigical information provided from TMUH, included diagnosis, histological type, histological grade, pTNM Pathological Classification, stage, age and gender in respective tissue. F, female; M, male; AD, adenocarcinoma; SCC, squamous cell carcinoma; SCLC, small cell lung carcinoma; pN, pathologic lymph node status; pT, Primary Tumour; pM, Distant Metastasis.

Figure S6. Computational analysis reveals post-translational modification sites of PKR, OAS and IL-24. Post-transcriptional modification sites of (**A**) PKR, (**B**) OAS and (**C**) IL-24 are predicted by computational analysis. NetPhos (http://www.cbs.dtu.dk/services/NetPhos/) was used for Phosphorylation site prediction (red). NetNGlyc (http://www.cbs.dtu.dk/services/NetNGlyc/) was used for Glycosylation site prediction (box). GPS-Lipid (http://lipid.biocuckoo.org/webserver.php) was used for lipidation site prediction (green highlights). UbPred (http://www.ubpred.org/) was used for ubiquitination site prediction (yellow highlights).
